# Supplementary material for: Density Functional Theory, Chemical Reactivity, Pharmacological Potential and Molecular Docking of Dihydrothiouracil-Indenopyridopyrimidines with Human-DNA Topoisomerase II
Source: Int J Mol Sci. 2020 Feb 13;21(4):1253. doi: 10.3390/ijms21041253 (PMC7072893; doi:10.3390/ijms21041253)
Supplement: Supplementary file 1 [file ijms-21-01253-s001.pdf]

Supplementary material (Figures)

DFT, Chemical reactivity, pharmacological potential and Molecular Docking of dihydro thiouracil - indenopyridopyrimidines with human-DNA topoisomerase II

Mohamed E. Elshakre [1]*, Mahmoud A. Noamaan [1]*, H. M. Moustafa [1], Haider Butt [2,3]

[1] Chemistry Department, College of Science, Cairo University, Cairo, Egypt, 12613

[2] School of Mechanical Engineering, University of Birmingham, Birmingham B15 2TT, U.K.

[3] Department of Mechanical Engineering, Khalifa University, Abu Dhabi 127788, UAE

* Correspondence: elshakre@sci.cu.edu.eg; Noamaan@sci.cu.edu.eg

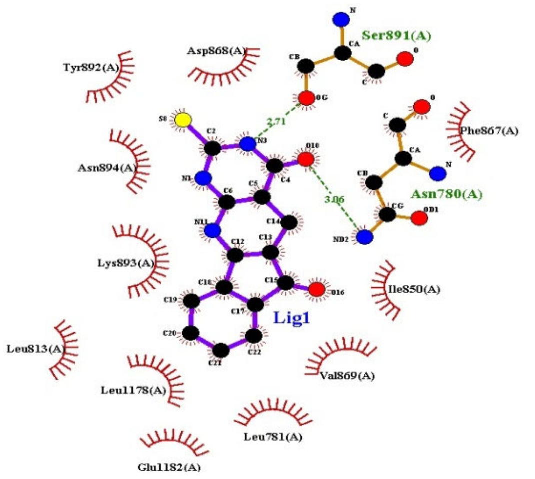

1H

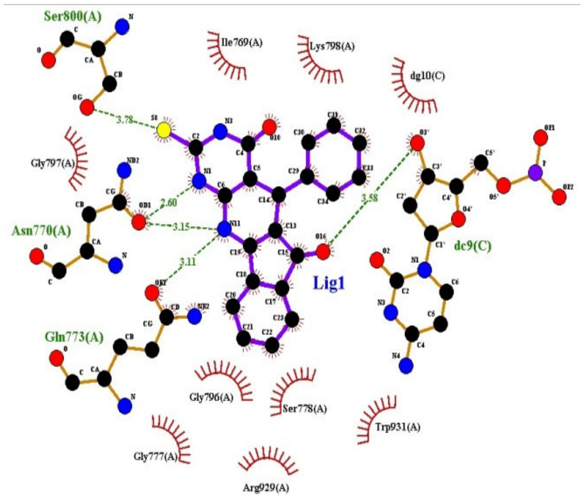

2H

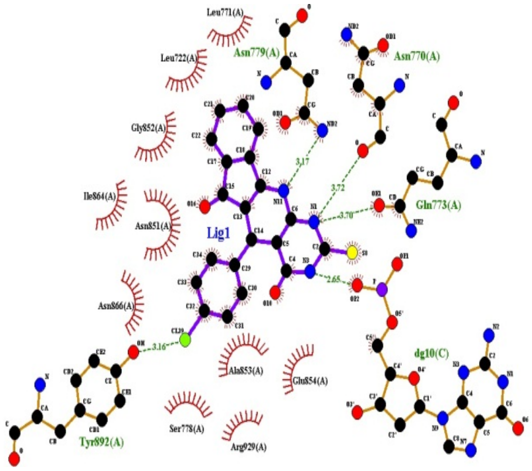

3H

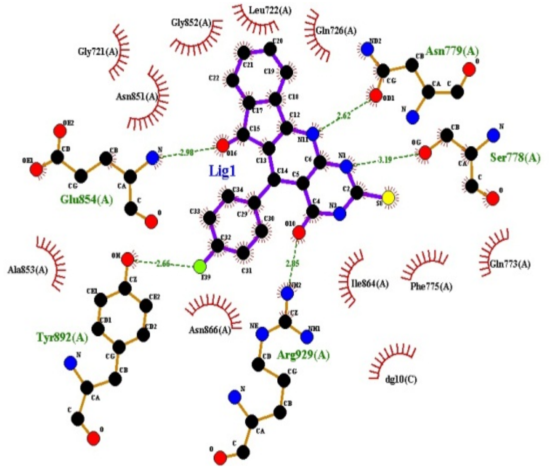

4H

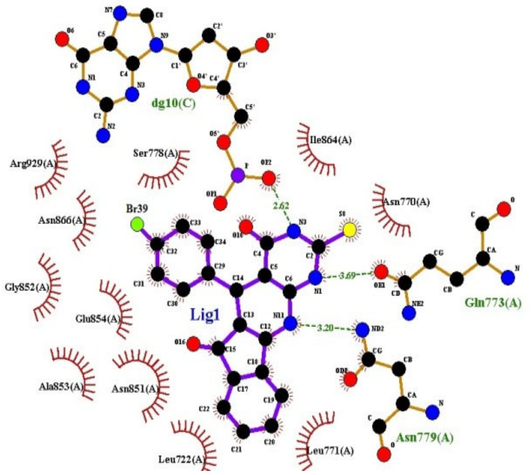

5H

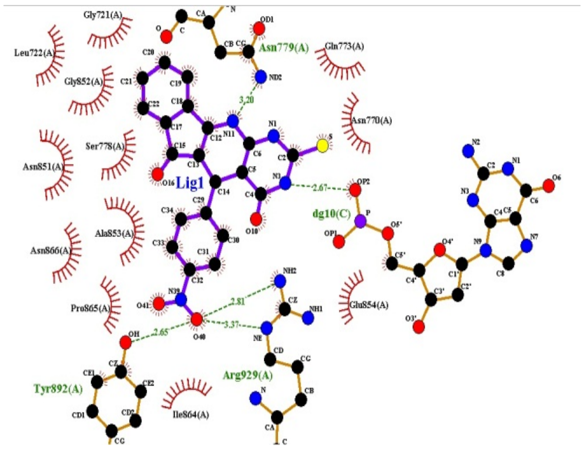

6H

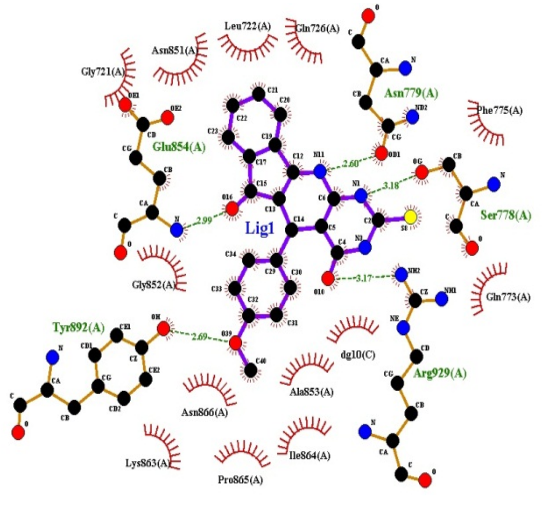

7H

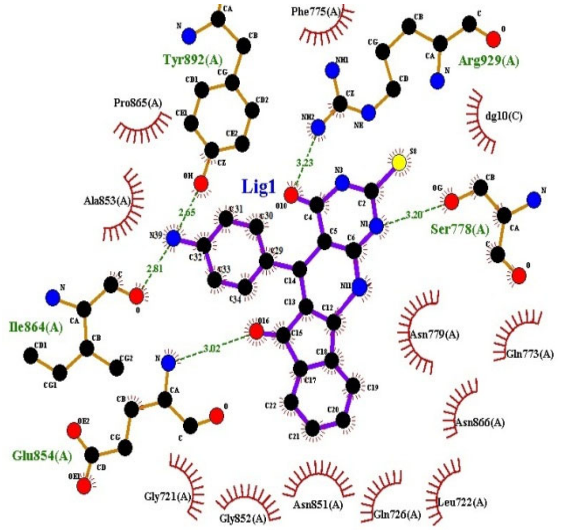

8H

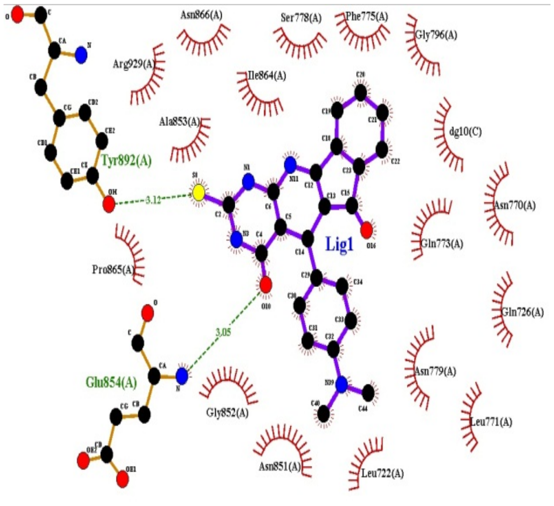

9H

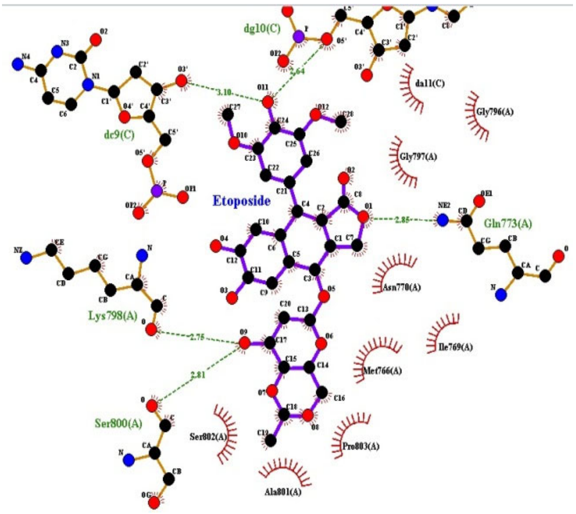

Etoposide

Figure S1. Two-dimensional binding sites scheme of the compounds of TUDHIPP **(1H-9H)** with human DNA topoisomerase IIα, **4fm9**

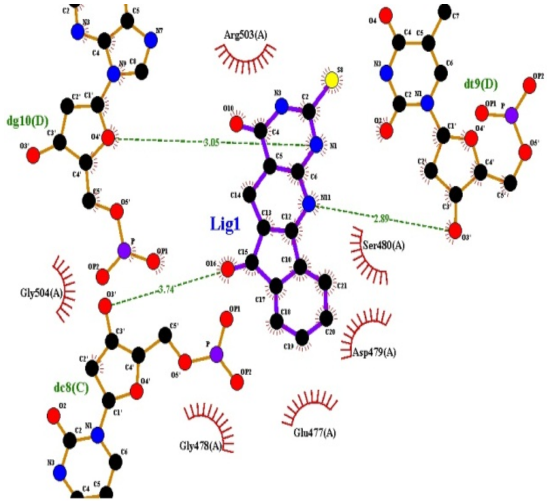

1H

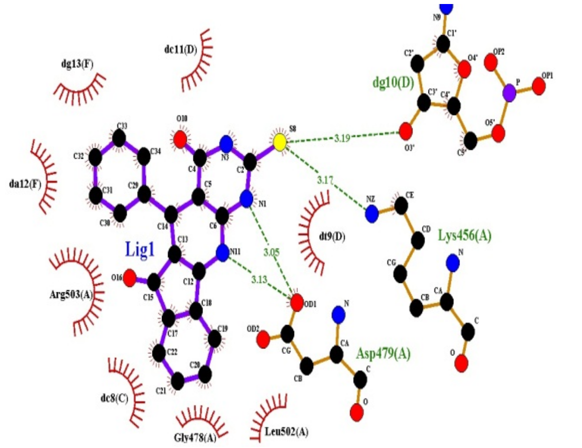

2H

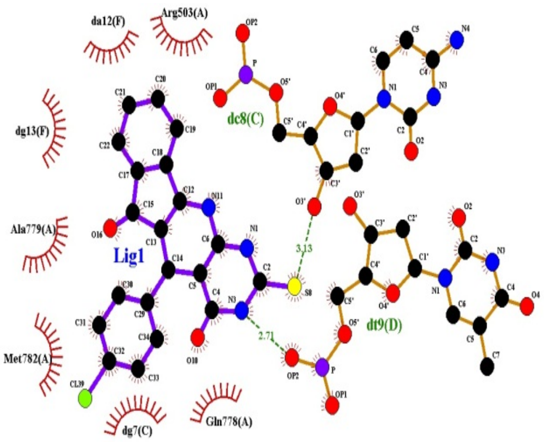

3H

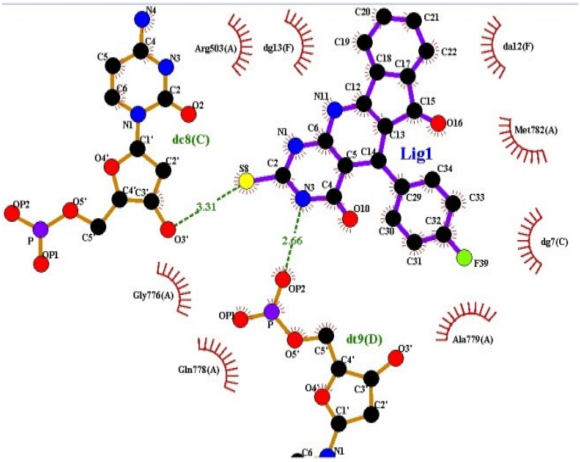

4H

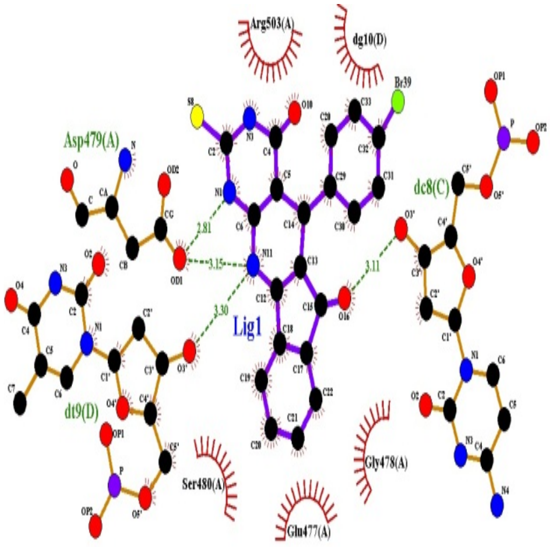

5H

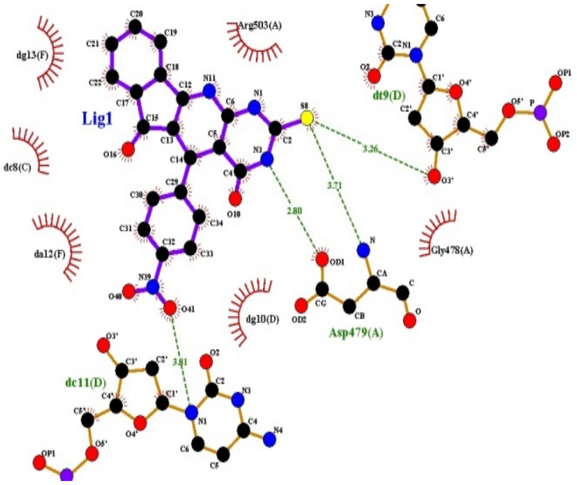

6H

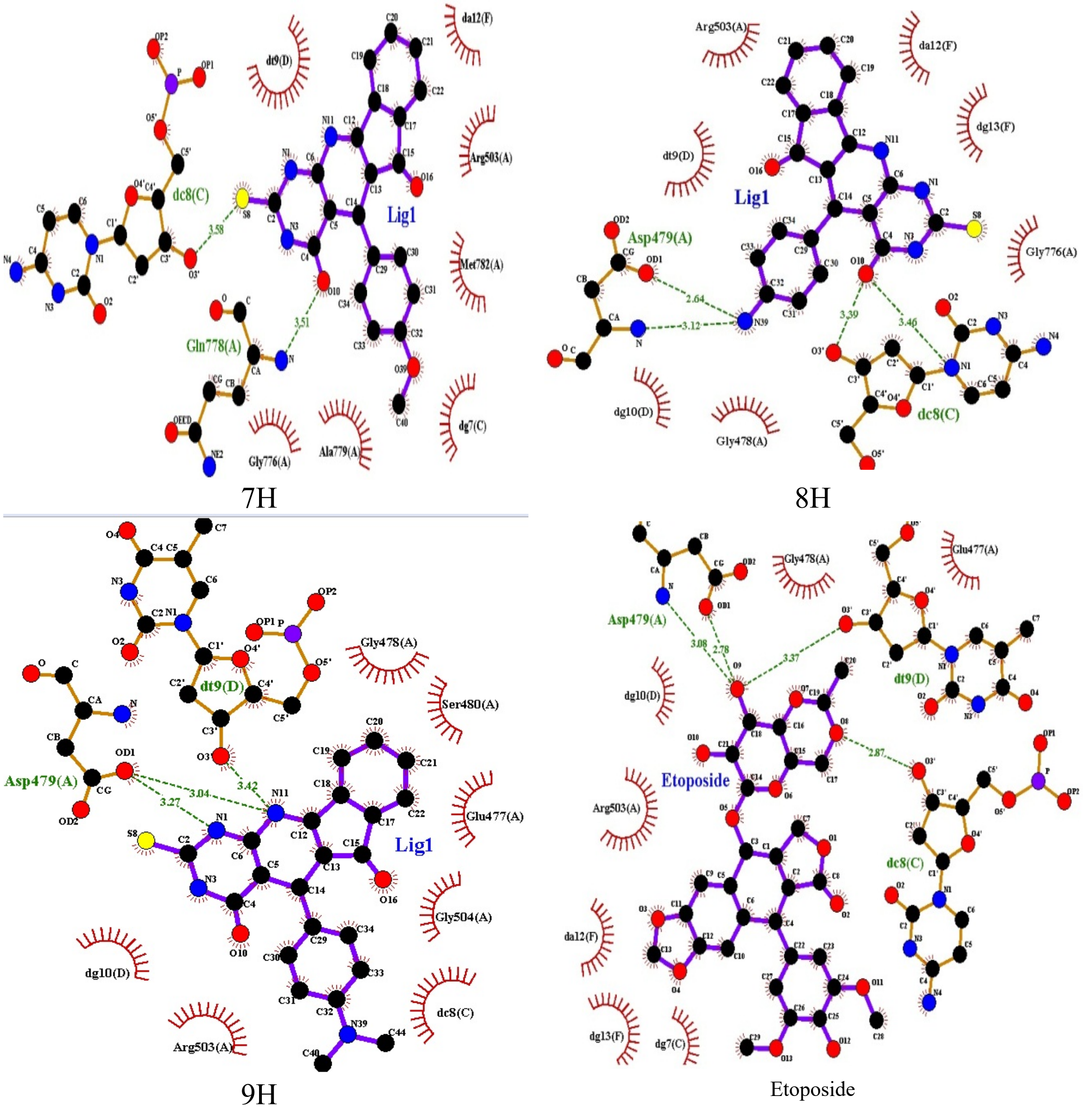

Figure S2. Two-dimensional binding sites scheme of the compounds of TUDHIPP **(1H-9H)** with human DNA topoisomerase II β, **<u>3qx3</u>**
